# Reliability and validity of time-use surveys in assessing 24-hour movement behaviors in adults

**DOI:** 10.1016/j.jesf.2025.03.003

**Published:** 2025-03-22

**Authors:** Nucharapon Liangruenrom, Kanyapat Suttikasem, Dyah Anantalia Widyastari, Danusorn Potharin, Piyawat Katewongsa

**Affiliations:** Institute for Population and Social Research, Mahidol University, Phutthamonthon, Nakhon Pathom, 73170, Thailand

**Keywords:** Behavioral epidemiology, Diary, Objective measurement, Self-report, Validation

## Abstract

**Background:**

Time-use surveys are considered a valid alternative for assessing physical activity (PA) and sedentary behavior (SB). The International Classification of Activities for Time-Use Statistics (ICATUS) has been widely adopted, as a standardized framework for categorizing time-use data. A classification system has been developed to classify ICATUS-based activities into sleep, SB, light PA (LPA), and moderate-to-vigorous PA (MVPA). This study aimed to evaluate the reliability and validity of ICATUS-based time-use classifications.

**Methods:**

Participants aged 18–59 years were recruited from five organizations located in the Bangkok metropolitian area (*n* = 220). The study was conducted from September to October 2022. Participants wore an accelerometer for ten consecutive days and completed two-day time-use diaries. The intraclass correlation coefficient (ICC) was calculated to assess test-retest reliability between the first and second entries of time-use surveys, as well as for accelerometer data. Validity was assessed by comparing the two time-use surveys with corresponding accelerometer data using Spearman correlations.

**Results:**

Test-retest reliability showed strong absolute agreement in the average time-use estimates for sleep and SB, with ICCs of 0.80 and 0.83, respectively. Moderate agreement was observed for LPA (ICC = 0.71) and MVPA (ICC = 0.51). Moderate validity correlations were found for SB, while LPA showed weak correlations, and MVPA results were inconsistent.

**Conclusion:**

ICATUS-based time-use data demonstrated strong reliability and moderate validity for SB, and moderate reliability and low validity for PA in working adults. The classification system appears to be a verified tool, supporting its applicability of time-use data, particularly in developing countries.

## 1 Background

Physical activity (PA) has beneficial effects on a range of health outcomes, including mortality reduction, chronic disease prevention and quality of life improvement.^1^^,^^2^ In contrast, sedentary behavior (SB) increases the risks of mortality, disability, cognitive decline, and depression.^3^^,^^4^ Monitoring these behaviors in a population is, therefore, crucial for health promotion.^5^ A variety of measurement tools have been developed for the surveillance of these behaviors. Self-reported questionnaires are a primary method, with several options available for use.^6^ The International Physical Activity Questionnaire (IPAQ) and Global Physical Activity Questionnaire (GPAQ) are the most widely used and recognized as valid self-report tools for estimating PA in multiple countries.^7^, ^8^, ^9^ However, these questionnaires have limitations due to recall bias and subjectivity, necessitating cautious use.^10^ Additionally, as SB is a distinct behavior, a need for a valid assessment to distinguish between PA and SB (e.g. sitting, lying, or standing) has arisen.^11^ Device-based measures, such as accelerometers, were subsequently introduced as a reliable and valid tools for both PA and SB.^12^

Emerging evidence suggests that movement behaviors - including PA, SB, and sleep--are integral and co-dependent components of a 24-h day.^13^ When examining any of these behaviors, it is essential to consider the full composition of the day. Accordingly, accelerometers, which measure bodily movements and detect activity intensities, have been increasingly used to ensure valid 24-h data in most studies on movement behaviors.^14^ However, devices, such as accelerometers, pose challenges, particularly for large-scale epidemiological studies in low- and middle-income countries, where simplicity and affordability are significant concerns.^12^ In such cases, self-report measures, such as time-use survey, are preferred. Additionally, behaviors assessed solely by objective measures lack detail on the domains and settings in which they occur - information that is crucial for developing effective interventions.^15^ This underscores the importance for incorporating subjective measures to assess behaviors across different domains and settings.

Time-use surveys are considered a valid alternative for assessing PA and SB.^16^^,^^17^ These surveys record spontaneous and continuous activities, allowing for detailed investigations of behaviors and potentially minimizing reporting biases, such as overestimating PA.^16^ Among various methods for collecting time-use data, the self-report diary is widely used.^18^ This method requires participants to document their daily activities in their own words at regular intervals, offering a rich qualitative dataset that can be systematically analyzed. However, a key challenge lies in standardizing and classifying these data to enable meaningful comparisons across populations and studies.

The International Classification of Activities for Time-use Statistics (ICATUS), developed by the United Nations Statistics Division, has been widely adopted, particularly in developing countries, as a standardized framework for categorizing time-use data.^19^ ICATUS organizes activities into a hierarchical structure with categories that range from broad domains, such as employment and learning, to more specific subcategories such as socializing and communication. Since its introduction in 1995, ICATUS has been employed in national time-use surveys across approximately 50 countries.^19^ Additionally, a previous study developed a classification system that maps ICATUS-based activities into movement behaviors including sleep, SB, light PA (LPA), and moderate-to-vigorous PA (MVPA).^20^ This system allows researchers to transform qualitative, self-reported time-use data into quantitative measures that facilitate systematic analysis across various dimensions. To maximize the utility of time-use data, it is recommended to assess the reliability and validity of PA and SB estimates when classified using this ICATUS-based classification.^20^

Until recently, only the Australian time-use survey and the Harmonised European Time Use Study (HETUS) classifications have been validated for assessing PA and SB.^16^^,^^17^ Given the increasing interest in producing nationally representative time-use data in both developing and developed countries, validating an international classification, such as ICATUS, may enhance the applicability and availability of time-use data for movement behavior epidemiology. Additionally, several countries that have conducted time-use surveys using ICATUS would perfer to maintain consistency in classification, it is, therefore, essential to establish its reliability and validity for estimating PA and SB. This study aims to address this gap by evaluating the test-retest reliability and concurrent validity of applying the classification system to ICATUS-based time-use data, ensuring its effectiveness in categorizing activities into movement behaviors.

## 2 Methods

### 2.1 Participants

According to Walter, Eliasziw and Donner,^21^ the minimum sample size was calculated using a minimum intraclass correlation coefficient (ICC) of 0.70, an expected ICC of 0.80, a 95 % significance level, 80 % power, and two repetitions of observation. A minimum of 148 participants was required.^22^ This study used a convenience sampling to recruit working adults from office-based workplaces located in the Bangkok metropolitian area. Five organizations accepted the invitation to allow their staff to take part in the study. Participation was open without restrictions on job titles or specific roles to all employees aged 18–59 years with Thai nationality, and volunteered to participate. The study was conducted from September to October 2022. A total of 247 workers volunteered to join and gave written informed consent prior to participation in the study. Of these, 220 employees (89 %) completed the study with complete questionnaire data, while 158 participants (64 %) had complete questionnaire and accelerometer data. The study protocol was approved by the Institutional Review Board of the Institute for Population and Social Research (IPSR-IRB) (COA. No. 2022/05–129).

### 2.2 Measurement and data collection

To assess whether movement behaviors derived from ICATUS-based self-reported diaries produce consistent and valid estimates when compared with accelerometer data, we utilized a structured questionnaire consisting of three components: personal information on sociodemographic characteristics, a 24-h time-use diary, and an accelerometer log. Socio-demographic data were collected through interviews conducted by the research team, capturing characteristics of participants, including sex, age, highest education level, marital status, monthly income, and predominant posture at work. Participants’ height and weight were also measured by a stadiometer (Seca 217, Seca, Hamburg, Deutschland) and a body composition monitor (BC-587, Tanita Corp, Japan), respectively to calculate their body mass index (BMI).

For the 24-h time-use diary, participants recorded their activities over four days, which were distributed across two time periods: days 2 and 3 (referred to as the 1st time-use survey) and days 9 and 10 (the 2nd time-use survey). These two periods corresponded to the same days of the week, with 55.2 % of entries from weekdays and 44.8 % from weekend days. On each recording day, participants recorded activities in 10-min intervals from 00.00 to 24.00 h, describing their primary activities in their own words, and when engaged in multiple activities simultaneously, also reported secondary activities. Additional information were collected, including the similarity of activities between the two periods (day 2 vs. day 9 and day 3 vs. day 10), the purpose of activities, their location, and the mode of transport used (if applicable).

The accelerometer log complemented the time-use diary by providing device-based measurement of movement behaviors. Participants wore an accelerometer (ActiGraph GT9X Link; ActiGraph, LLC., Pensacola, FL, USA) for ten consecutive days during their waking hours. Following standard recommendations for assessing PA in adults under free-living conditions, the accelerometer was worn on the right hip, attached on a waistband.^23^ Participants were asked to wear the device upon waking and remove it only when engaging in water-based activities and during bedtime. Although accelerometers can track sleep, it is recommended to be worn on the wrist for more accurate sleep measurements. To minimize participant discomfort and reduce burden, wearing the device on the hip was prioritized. Participants also logged the start and end times of accelerometer use each day, along with reasons for non-wear. Self-reported wake and bed times were found to be highly associated with accelerometer-determined waking hours,^24^ supporting the validity of the combined data collection approach.

With a limited number of accelerometer available, data collection was conducted one workplace at a time. At each site, the research team provided instructions on time-use diary recording, accelerometer usage, and the accelerometer log. During the study period, the research team provided reminders to wear the device and record data in the accelerometer log and time-use diary through phone calls or LINE, an instant messaging application widely used in Thailand. This approach aimed to minimize missing data while preserving the integrity of self-reported response. Participants could also ask questions or report difficulties regarding the study through these communication channels. On day 11, the research team visited each work site to collect the devices, logs and diaries, and ensured the data was complete.

### 2.3 Data processing

For the time-use survey, all activities recorded in the diary were coded according to the Trial International Classification of Activities for Time-Use Statistics (ICATUS), a five-level hierarchical classification of 15 major divisions of activities.^25^ The coding process was conducted by a team of trained raters who underwent an initial workshop to ensure familiarity with ICATUS codes. These ICATUS-based codes were then categorized into sleep, SB, LPA, and MVPA using the classification system developed in a previous study.^20^ The method used to develop the standardized criteria for classifying ICATUS-based activities can be found elsewhere.^20^ The entire process, from free-text entries to ICATUS-based codes and final categorization, was conducted by raters and rechecked by another team member. Regular meetings were held to address any ambiguities and clarify coding rules. These measures ensured the robustness and consistency of the time-use data for subsequent analyses.

For some work- and travel-related activities that were broadly recorded as working and travelling, we linked these activities with additional information available from the questionnaire to assign the movement behaviors as suggested in previous studies.^20^^,^^26^^,^^27^ For work-related activities, we used the question that asked participants to identify their predominant posture at work as mostly sitting, mostly standing, mostly walking, or mostly using labor. These postures were assigned with corresponding MET values available in the Compendium of Physical Activities.^28^ For travel-related activities, respondents had to provide the mode of travel when their activities were related to travelling. These modes were linked with METs assigned in previous studies based on the Compendium of Physical Activities.^26^^,^^28^^,^^29^ Once all ICATUS-based activities were assigned, the average time spent in movement behaviors was calculated. Time spent in each behavior (i.e. sleep, SB, LPA, and MVPA) of days 2 and 3 was used to calculate the average daily time for the 1st time-use survey, and that of days 9 and 10 for the 2nd time-use survey.

For accelerometers, the data recorded in the device was downloaded and processed using the ActiLife version 6.13.4 software (ActiGraph, LLC., Pensacola, FL, USA). We used a sampling rate of 50 Hz with 1-min epoch. If participants were not wearing the accelerometer for at least 90 consecutive minutes (the intensity count was equal to zero, with an allowance of up to a 2-min interval of <100 counts per minute), it was considered as non-wear time.^30^ To be included in the analysis, the accerelometry data of collection days 2, 3, 9, and 10 had to be at least 10 h/day of wear time. The cutoff points for SB (<100 counts per minute), LPA (100–2019 counts per minute), and MVPA (≥2020 counts per minute) were used to classify each 1-min epoch.^31^ The valid accelerometer count data of days 2 and 3 was used to calculate the average time of the 1st time-use accelerometer data, and that of days 9 and 10 for the 2nd time-use accelerometer data.

### 2.4 Data analysis

We analyzed the data using the R program (R Foundation for Statistical Computing, Vienna, Austria) with the “spearmanCI”,^32^ “irr”,^33^ “psych”,^34^ and “dplyr”^35^ packages. Frequency and percentage were used to describe the characteristics of the study sample, including sex, age, highest education level, marital status, monthly income, and predominant posture at work. These variables were included to provide a comprehensive overview of the participants and were not used in the primary analyses, which focused on the reliability and validity of time-use estimates. Means and standard deviations (SDs) were used to present the amount of time spent in each movement behavior estimated by the time-use surveys and accelerometers.

For the test-retest reliability analysis, two-way mixed-effects ICCs and their 95 % confidence intervals (CIs) were calculated to assess the absolute agreement and consistency of participants’ average estimates between the 1st and 2nd time-use surveys, as well as between the 1st and 2nd accelerometer-based measurements. Participants also rated the similarity of their activities between the two periods of time-use data collections (Day 2 vs Day 9; Day 3 vs Day 10) on a scale from 1 to 10 (1 = no difference at all; 10 = totally different). Separate analyses for the tes-retest reliability were conducted for i) those who reported similar activities (scores of 1–4), ii) those who reported different activities (scores of 5–10), and iii) the total sample.^16^ ICCs were classified as excellent (≥0.90), good (0.75–0.89), moderate (0.50–0.74), or weak (<0.5).^36^

For the validity test, estimates of SB, LPA, and MVPA from the two time-use surveys were compared with those from the corresponding accelerometers using Spearman correlations. The validity was assessed separately for the 1st and 2nd entries. The correlations were interpreted as excellent (0.90–1.00), good (0.70–0.89), moderate (0.40–0.69), weak (0.10–0.39), or negligible (0.00–0.10).^37^

We conducted Bland-Altman analysis to assess the agreement between ICATUS-based time-use survey and accelerometer-derived measures of SB, LPA, and MVPA. The mean differences (bias) and 95 % limits of agreement (LOA) were calculated to evaluate the degree of systematic error. A regression analysis of the differences against the mean values was performed to determine proportional bias. A statistically significant slope (p < 0.05) indicated that the measurement discrepancy varied depending on activity levels.

## 3 Results

A total of 220 participants with complete test-retest reliability data for the time-use surveys were included in the analysis. Of these, 158 had complete accelerometer data and were thus included in the reliability test between accelerometers and validity analyses.

The majority of the participants were female and had a bachelor's degree. Most of them worked in a sittting posture. Approximately half of the participants were never married. Around 36 % of the employees had a moderate monthly income, while 35 % had a low income. Compared to the national average, this suggests that office workers in Bangkok tend to have higher wages.^38^ In our sample, 46.8 % had BMI of 25 kg/m^2^ or higher, which is comparable to the national average of 42.4 % among Thai adults.^39^ (Table 1).

**Table 1:** Characteristics of total participants (*n* = 220) and participants with accelerometer data (*n* = 158).

Variable	All sample *n* (%)	Sample with accelerometer data *n* (%)
**Sex**		
• Female	130 (59.1 %)	95 (60.1 %)
• Male	90 (40.9 %)	63 (39.9 %)

**Age** (median [IQR])	36.0 [14.0]	38.0 [14.7]

**Highest education level**		
• Primary school	1 (0.5 %)	1 (0.6 %)
• High school	9 (4.1 %)	6 (3.8 %)
• Diploma	12 (5.5 %)	8 (5.1 %)
• Bachelor degree	154 (70.0 %)	115 (72.8 %)
• Higher than bachelor degree	44 (20.0 %)	28 (17.7 %)

**Marital status**		
• Never married	118 (53.6 %)	81 (51.3 %)
• Married	88 (40.0 %)	65 (41.1 %)
• Not married, but lived together	9 (4.1 %)	9 (5.7 %)
• Divorced/separated	4 (1.8 %)	2 (1.3 %)
• Not identified	1 (0.5 %)	1 (0.6 %)

**Monthly income**^a^		
• Very low (≤10,000 baht)	3 (1.4 %)	2 (1.3 %)
• Low (10,001–20,000 baht)	77 (35.0 %)	48 (30.4 %)
• Moderate (20,001–40,000 baht)	80 (36.4 %)	57 (36.1 %)
• High (40,001–100,000 baht)	57 (25.9 %)	48 (30.4 %)
• Very high (>100,000 baht)	3 (1.4 %)	3 (1.9 %)

**Predominant posture at work**		
• Mostly sitting	181 (82.3 %)	130 (82.3 %)
• Mostly standing	1 (0.5 %)	1 (0.6 %)
• Mostly walking	23 (10.5 %)	17 (10.8 %)
• Mostly using labor	5 (2.3 %)	2 (1.3 %)
• Others	10 (4.5 %)	8 (5.1 %)

**Body mass index status**^b^		
• Underweight (<18.5 kg/m^2^)	7 (3.2 %)	6 (3.8 %)
• Normal (18.5–22.9 kg/m^2^)	73 (33.2 %)	52 (32.9 %)
• Overweight (23–24.9 kg/m^2^)	37 (16.8 %)	26 (16.5 %)
• Obese (25–29.9 kg/m^2^)	81 (36.8 %)	58 (36.7 %)
• Morbidly obese (≥30.0 kg/m^2^)	22 (10.0 %)	16 (10.1 %)

Abbreviation: IQR = interquartile range.

Note.

a 1 United States Dollar = 33.82 Thai Baht (as of January 29, 2025); the average monthly income in Thailand was approximately 15,000–20,000 (National Statistical Office of Thailand). .

b The prevalence of obesity (BMI of 25 kg/m^2^ or higher) among Thai adults was 42.4 % (The 6th National Health Examination 2019–2020). .

Based on participants' time-use surveys, participants spent approximately 44.1 % of their time in SB (mean = 634–636 min per day), followed by sleep around 36.3 % (mean = 519–526 min per day), LPA around 18.1 % (mean = 260–262 min per day) and MVPA around 1.5 % (mean = 19–23 min per day). For participants who engaged in similar activities across both assessment periods, the test-retest reliability analysis indicates a strong absolute agreement in the average time-use estimates for sleep and SB, with ICCs of 0.80 and 0.83, respectively. Additionally, we observed good consistency in the rankings of participants’ sleep and SB estimates between the two surveys.

In contrast, for LPA and MVPA, the analysis revealed moderate absolute agreement with ICCs of 0.71 and 0.51, respectively. Consistency in the rankings for LPA and MVPA estimates was also moderate.

When considering the total sample, the test-retest reliability suggests a moderate absolute agreement in average time-use estimates across the two assessments for sleep, SB, and LPA, with ICCs ranging from 0.60 to 0.72. The consistency of participants’ rankings for these behaviors was similarly moderate. However, for MVPA, both the absolute agreement and consistency were lower with an ICC of 0.40 (Table 2).

**Table 2:** Test-retest reliability for absolute agreement and consistency of all movement behaviors estimated in the first and second time-use surveys (*n* = 220).

Movement behavior	1st Time-use Survey (min/day)	2nd Time-use Survey (min/day)	Test-retest reliability for Absolute Agreement			Test-retest reliability for Consistency		
	Mean (SD)	Mean (SD)	ICC (95 % CI) (*n* = 85)^a^	ICC (95 % CI) (*n* = 67)^b^	ICC (95 % CI) (*n* = 220)^c^	ICC (95 % CI) (*n* = 85)^a^	ICC (95 % CI) (*n* = 67)^b^	ICC (95 % CI) (*n* = 220)^c^
**Sleep**	519 (106)	526 (124)	0.80 (0.69, 0.87)	0.48 (0.16, 0.68)	0.72 (0.64, 0.79)	0.79 (0.68, 0.87)	0.48 (0.15, 0.68)	0.72 (0.64, 0.79)
**SB**	636 (137)	634 (164)	0.83 (0.74, 0.89)	0.40 (0.02, 0.63)	0.70 (0.60, 0.77)	0.83 (0.74, 0.89)	0.39 (0.01, 0.63)	0.70 (0.60, 0.77)
**LPA**	262 (123)	260 (130)	0.71 (0.56, 0.81)	0.42 (0.05, 0.64)	0.60 (0.48, 0.70)	0.71 (0.56, 0.81)	0.42 (0.05, 0.64)	0.60 (0.48, 0.69)
**MVPA**	23 (33)	19 (34)	0.51 (0.24, 0.68)	0.27 (−0.19, 0.55)	0.40 (0.22, 0.54)	0.51 (0.24, 0.68)	0.27 (−0.19, 0.55)	0.40 (0.22, 0.54)

Abbreviations: SD = standard deviation; ICC = intraclass correlation coefficient; CI = confidence interval; SB = sedentary behavior; LPA = light physical activity; MVPA = moderate to vigorous physical activity.

a participants who reported similar activities between the two periods of time-use data collections (scores of 1–4).

b participants who reported different activities between the two periods of time-use data collections (scores of 5–10).

c the total sample.

Based on accelerometer data, participants spent most of their waking hours in SB (mean = 603–634 min per day), followed by LPA (mean = 240–242 min per day) and MVPA (mean = 12–14 min per day). For participants who reported performing similar activities during both assessments, the test-retest reliability analysis shows a strong absolute agreement in average time-use estimates across the 1st and 2nd accelerometer measurements for SB, LPA, and MVPA, with ICCs of 0.76, 0.82, and 0.81, respectively. Additionally, we observed good consistency in the rankings of participants’ SB, LPA, and MVPA estimates between the two accelerometer measurements.

In the total sample, as well as among participants who reported differences in activities between the two assessments, the test-retest reliability indicated a moderate absolute agreement in average time-use estimates for SB, LPA, and MVPA, with ICCs ranging from 0.62 to 0.72. Similarly, moderate consistency was found in the rankings of participants’ SB, LPA, and MVPA estimates, with ICCs ranging from 0.63 to 0.73 (Table 3).

**Table 3:** Test-retest reliability for absolute agreement and consistency of all movement behaviors estimated in the first and second accelerometer-based measurements (*n* = 158).

Movement behavior	1st Time-use Accelerometer (min/day)	2nd Time-use Accelerometer (min/day)	Test-retest reliability for Absolute Agreement			Test-retest reliability for Consistency		
	Mean (SD)	Mean (SD)	ICC (95 % CI) (*n* = 64)^a^	ICC (95 % CI) (*n* = 60)^b^	ICC (95 % CI) (*n* = 158)^c^	ICC (95 % CI) (*n* = 64)^a^	ICC (95 % CI) (*n* = 60)^b^	ICC (95 % CI) (*n* = 158)^c^
**SB**	634 (131)	603 (151)	0.76 (0.60, 0.85)	0.72 (0.54, 0.83)	0.71 (0.60, 0.79)	0.76 (0.60, 0.85)	0.73 (0.54, 0.84)	0.72 (0.62, 0.80)
**LPA**	242 (78)	240 (82)	0.82 (0.71, 0.89)	0.67 (0.45, 0.80)	0.69 (0.57, 0.77)	0.82 (0.71, 0.89)	0.67 (0.45, 0.80)	0.69 (0.57, 0.77)
**MVPA**	14 (15)	12 (16)	0.81 (0.68, 0.88)	0.62 (0.37, 0.77)	0.67 (0.55, 0.76)	0.81 (0.68, 0.88)	0.63 (0.38, 0.78)	0.68 (0.56, 0.76)

Abbreviations: SD = standard deviation; ICC = intraclass correlation coefficient; CI = confidence interval; SB = sedentary behavior; LPA = light physical activity; MVPA = moderate to vigorous physical activity.

a participants who reported similar activities between the two periods of time-use data collections (scores of 1–4).

b participants who reported different activities between the two periods of time-use data collections (scores of 5–10).

c the total sample.

For the validity tests, estimates recorded in the 1st and 2nd surveys and accelerometers indicate moderate correlations for SB (rho = 0.40 and 0.45, respectively), and weak correlations for LPA (rho = 0.37 and 0.38, respectively). For MVPA, we found a moderate correlation for the first time entry (rho = 0.40), and a weak correlation for the second time entry (rho = 0.32). (Table 4).

**Table 4:** Concurrent validity tests of sedentary behavior, light physical activity, and moderate to vigorous physical activity estimated in the time-use surveys and accelerometers (*n* = 158).

Movement behavior	1st Time-use Survey (min/day)	1st Time-use Accelerometer (min/day)	Spearman's correlation (Survey 1 vs. Accelerometer 1)	2nd Time-use Survey (min/day)	2nd Time-use Accelerometer (min/day)	Spearman's correlation (Survey 2 vs. Accelerometer 2)
	Mean (SD)	Mean (SD)	rho (95 % CI)	Mean (SD)	Mean (SD)	rho (95 % CI)
**SB**	644 (140)	634 (131)	0.40 (0.26, 0.54)	641 (167)	603 (151)	0.45 (0.32, 0.58)
**LPA**	263 (125)	242 (78)	0.37 (0.23, 0.51)	256 (134)	240 (82)	0.38 (0.24, 0.52)
**MVPA**	23 (33)	14 (15)	0.40 (0.26, 0.54)	18 (34)	12 (16)	0.32 (0.16, 0.47)

Abbreviations: SD = standard deviation; CI = confidence interval; SB = sedentary behavior; LPA = light physical activity; MVPA = moderate to vigorous physical activity.

Bland-Altman analysis showed that the mean difference (bias) between time-use survey and accelerometer estimates ranged from −1.3 to +26.9 min across all activity levels (Table 5). The LOA were widest for SB and narrowest for MVPA. No significant proportional bias was detected for SB, Most data points fell within the LOA (see full Bland-Altman plots in Supplementary File 1).

**Table 5:** Mean differences, 95 % limits of agreement, and proportional bias in Bland-Altman analysis comparing movement behaviors estimated in the time-use surveys and accelerometers (*n* = 158).

Measurement period	Movement behavior	Mean Difference (Bias)	Upper LOA	Lower LOA	Proportional Bias (p-value)
1st comparisons (Days 2 vs. 9)	SB	−1.3 min	+297.7	−300.2	0.201
	LPA	+20.5 min	+252.3	−211.3	<0.001∗
	MVPA	+9.9 min	+72.8	−52.9	<0.001∗
2nd comparisons (Days 3 vs. 10)	SB	+26.9 min	+384.7	−330.8	0.427
	LPA	+17.9 min	+268.5	−232.7	<0.001∗
	MVPA	+6.2 min	+68.5	−56.2	<0.001∗

Abbreviations: SD = standard deviation; CI = confidence interval; SB = sedentary behavior; LPA = light physical activity; MVPA = moderate to vigorous physical activity; LOA = limits of agreement; ∗significant level (*p* < 0.05).

## 4 Discussion

The ICATUS-based time-use survey demonstrated strong reliability for sleep and SB, and moderate reliability for LPA and MVPA. Regarding concurrent validity, the ICATUS-based time-use survey showed moderate validity for SB, with no proportional bias, and lower validity for LPA and MVPA, with significant proportional bias. The validity of MVPA estimates yielded mixed results. The first comparison between the time-use survey and accelerometer data showed moderate validity, whereas the second comparison indicated lower validity. These findings suggest that while the ICATUS-based time-use classification provide strong estimates for SB, caution is needed when interpreting LPA and MVPA.

Although device-based measurements are often considered more accurate for estimating PA and SB, self-report assessments remain the most practical method for collecting movement behaviors data, particularly in large observational studies.^12^ Our findings suggest that time-use data can be a better alternative to traditional PA questionnaires. The reliability of the ICATUS-based time-use survey demonstrated moderate to strong correlations, consistent with the reliability reported for PA questionnaires such as the IPAQ and GPAQ in the previous studies (correlation coefficients ranging from 0.58 to 0.89).^8^^,^^10^^,^^40^ When the ICATUS-based time-use survey was evaluated against accelerometer data, the validity of SB estimates from the survey showed somewhat higher correlations (0.40–0.45) compared to sitting or SB estimates from PA questionnaires assessed using accelerometers (0.12–0.38).^8^^,^^10^^,^^40^ Although the validity of LPA and MVPA estimates from the time-use survey was lower, the results are comparable to those of existing PA questionnaires. Specifically, the validity of LPA estimates (0.37–0.38) was higher than that observed in self-reported PA questionnaires (0.08–0.22),^10^ while the validity of MVPA estimates (0.32–0.40) was similar to that of several PA questionnaires (0.34–0.48).^10^^,^^40^

SB appeared to be the most reliable and valid estimate in the time-use survey within the context of this study, aligning with findings from the Australian time-use survey, which reported high reliability and validity for non-occupational SB (0.57–0.59).^16^ Notably, the validity of SB estimates derived from diaries was more favorable compared to those obtained from questionnaires, with correlation coefficients of 0.63 and 0.35, respectively.^15^ The non-significant proportional bias also suggests that agreement between the time-use survey and accelerometer remains stable across different levels of sedentary time. This indicates that recording SB-related activities tends to be clear and straightforward. Specifically, in this study, the majority of participants reported sitting as their predominant posture at work, which involved fixed and predictable periods. Given that participants were recruited from office-based workplaces, their work environment may have contributed to the consistency in SB reporting. Additionally, drawing on recommendations from previous studies to include detailed breakdowns of activities within broad categories such as working time, we encouraged participants to record activities as precisely as possible.^20^^,^^26^^,^^41^ For example, many participants specified tasks such as attending online meetings, working on a computer, or packing, rather than simply recording “working” in general. This approach enhanced the accuracy of the SB assessment.

LPA and MVPA estimates from the time-use survey appeared to have lower reliability compared to SB. This discrepancy might be attributed to limitations in the coding system used for time-use data. Notably, LPA and MVPA estimates derived from the survey were higher than those obtained from accelerometers. One key issue is that most ICATUS activities were assigned unweighted MET values, calculated from the list of matched Compendium activities.^20^ This approach assumes that each activity is of uniform intensity, without accounting for the varying time spent on tasks of differing intensities within the same activity. For example, the activity “household cleaning” is classified as MVPA, yet it can involve various tasks with differing intensities, such as light dusting, moderate vacuuming, and vigorous mopping. If a participant spent 30 min cleaning, primarily dusting with a brief period of mopping at the end, the entire half-hour would be categorized as MVPA rather than 20 min of LPA and 10 min of MVPA.

Additionally, the intensity of physical activities may increase over time. For example, washing a car might start with light effort during the first 10 min, progressing to moderate effort in the final 20 min, yet the entire activity would be classified as MVPA. These varying tasks, however, are not reflected in the unweighted MET values, which simply assign an average MET for the entire activity. As a result, this can potentially lead to an overestimation of LPA and MVPA. A more precise estimation could be achieved by calculating weighted averages, where the weights correspond to the proportion of time spent on each task within the activity, or ideally, to the representation of these activities in the time use of a specific population.^20^ This highlights the complexity and variability of human activities.^42^ Given these limitations and the generic nature of the estimates, time-use data is generally more suitable for population-level surveillance than individual assessment.^16^^,^^42^

It is also important to note that the test-retest reliability results showed that both the time-use survey and accelerometer exhibited identical ICCs for absolute agreement and consistency within their respective methods, indicating stable measurements across repeated assessments. However, ICCs differed between the two tools, suggesting methodological differences in how movement behaviors were captured. The self-report time-use survey relies on participants' recall and interpretation of activities, which may introduce recall biases or classification differences compared to the accelerometer's objective measurement of movement. Additionally, certain activities such as upper-body movements, standing, or brief postural shifts may be recorded differently between self-reports and hip-worn accelerometer data.^43^ These differences highlight the importance of considering measurement tools when interpreting movement behavior data.

The nature of MVPA activities may have also contributed to differences in validity observed between the first and second comparisons. Hip-worn accelerometers are well-suited for detecting ambulatory activities such as walking and running but may underdetect non-ambulatory activities of daily living such as housework.^43^ If participants engaged in a higher proportion of such activities during one measurement period, this could lead to discrepancies in MVPA estimates between time-use survey and accelerometer data. Although the study design ensured that the two time-use surveys were conducted on the same days of the week, external factors such as weather conditions, variations in daily routines, or social obligations might have led to different activity patterns. These fluctuations could explain why validity differed across time points.

As we also observed proportional bias for LPA and MVPA, this suggests that the difference between the ICATUS-based time-use survey and accelerometer estimates varies depending on the magnitude of the activity. This bias may be attributed to underestimation by accelerometers and recall challenges in self-reported time-use surveys. However, it is important to note that the Bland-Altman analysis also showed that most data points remained within the LOA. This supports an overall utility of the ICATUS-based classification system for estimating movement behaviors.

For sleep, the current study also suggests that sleep estimates from the time-use survey demonstrate strong reliability, with correlation coefficients around 0.80, compared to those from a questionnaire, which ranged from 0.59 to 0.61 in a recent study.^44^ This finding aligns with previous research, indicating that sleep durations assessed through diaries generally produce more reliable estimates than those obtained from questionnaires.^45^ Additionally, earlier studies have shown that self-reported sleep duration from questionnaires tends to overestimate objectively measured sleep.^46^^,^^47^ However, since we did not objectively collect sleep hours in this study, comparisons between sleep estimates from the time-use survey and those from accelerometer data could not be made. The observed differences in reliability and validity may stem from both the characteristics of the ICATUS-based classification system and the specific attributes of our study participants. ICATUS categorizes activities based on predefined codes, which may not always align perfectly with movement behavior classifications. For example, certain activities that involve both SB and LPA such as desk work with intermittent walking may be coded under a single ICATUS category, potentially affecting classification accuracy. Additionally, subject-dependent factors, such as occupational routines, recall ability, and interpretation of activity categories, could influence the reported time-use data. While the classification system itself provides a standardized framework, variations in how individuals recall and categorize their activities may contribute to discrepancies in movement behavior estimates.

Consistent with previous validation studies, we found that time-use data demonstrate good validity as a self-assessment tool for estimating PA and SB when compared to accelerometer data.^16^^,^^17^ The ICATUS-based classification system appears to be a verified tool, providing estimates of sleep, SB, LPA, and MVPA.^20^ This system uses metabolic equivalent (MET) values from the Compendium of Physical Activities.^28^ These MET values have also been linked in several other time-use surveys, such as the American Time-Use Survey (ATUS),^26^ Statistics Canada's General Social Survey on Time Use (GSS-TU)^41^ and the Halifax STAR time diary.^48^ Validating these estimates enhances the assessment of PA and SB, allowing researchers to capitalize on extensive existing and future time-use data. To date, several studies have used MET summaries assigned to time-use activities to examine population trends and time spent across all domains of SB, LPA, and MVPA.^49^, ^50^, ^51^, ^52^ The current validation of ICATUS should encourage further research, deepening our understanding of movement behaviors.

## 5 Strengths and limitations

The current study has several strengths, including the use of an accelerometer as a reference for validating ICATUS-based activities classified into SB, LPA, and MVPA. Additionally, the longer observation periods, at least seven days of accelerometer use and two days of time-use diaries, are optimum for capturing individual's activity patterns.^16^^,^^53^ We conducted two-day time-use surveys on two separate occasions (four days in total), with each pair of days proportionally distributed across the week. This was complemented by the collection of 10-day accelerometer data, allowing for double validation and improving the comparability of SB, LPA, and MVPA estimates from both assessments.

However, there are some limitations to consider. First, our study sample consisted of Thai urban working adults, which may limit the generalizability of the results to the broader adult population or different contexts. Second, participants were instructed to remove the accelerometer during sleep and water-based activities. This non-wear time could lead to missed activities recorded in the device and potentially result in an underestimation of concurrent validity. Third, the time-use survey was self-administered, with participants receiving instructions on how to record their activities, as well as written guidelines and reminders from the research team during data collection. Despite these efforts, there is a risk of errors due to varying levels of participant engagement and recall bias. Finally, given the nature of real-world activities, day-to-day fluctuations in behaviors may influence reliability estimates and complicates the interpretation of test-retest reliability. Future studies may consider integrating multiple methods to better isolate tool-specific reliability from behavioral variability for a more comprehensive assessment.

## 6 Conclusion

The ICATUS-based time-use data demonstrated strong reliability for assessing sleep and SB, moderate reliability for LPA and MVPA, and moderate validity for SB but lower validity for PA measures in working adults. Our findings highlight the potential of time-use surveys as a preferable alternative to traditional PA questionnaires, especially for population-wide studies. Since ICATUS is used in multiple countries, particularly in developing regions, the findings may be informative for other contexts where ICATUS-based time-use surveys are conducted. However, further research is needed to examine how these results translate to different cultural and economic settings. Future validation studies should also focus on a comprehensive assessment of 24-h movement behaviors to further validate and expand the applicability of these findings.

## Ethics approval and consent to participate

The study was approved by the Institutional Review Board of the Institute for Population and Social Research (IPSR-IRB), Mahidol University (COA. No. 2022/05–129). All participants were informed about the study processes and their rights. If they agreed, they would provide written consent before they participated in the research.

## Availability of data and materials

The datasets without confidential data is available from the corresponding author upon a reasonable request and with written letter from an authorised organization.

## Author statement

All authors read, agreed with the content, and approved the final draft manuscript. The manuscript is original and is not under consideration for publication elsewhere. We would be very grateful if you would consider the manuscript for publication in your journal.

Sincerely yours,

Nucharapon Liangruenrom, Kanyapat Suttikasem, Dyah Anantalia Widyastari, Danusorn Potharin, Piyawat Katewongsa.

## Funding

This research is part of the “Development of Academic Tools and Evidence to Support Policy Design and Decision Making in Physical Activity Promotion to Reduce NCDs” project funded by 10.13039/501100009061Thai Health Promotion Foundation (Grant No. 65-00-0258; Project No. 65–00345).

## Declaration of competing interest

Each author has contributed to the manuscript. NL and PK conceptualised the study. NL, DAW and DP conceptualised the data collection approach. NL and KS collected the data. NL conducted the data management and analysis of the datasets. NL drafted the manuscript. NL, KS, DAW, DP and PK reviewed and edited the manuscript. All authors read and approved the final draft.

This study is part of the “Development of Academic Tools and Evidence to Support Policy Design and Decision Making in Physical Activity Promotion to Reduce NCDs” project funded by Thai Health Promotion Foundation (Grant No. 65-00-0258; Project No. 65–00345).

The funder did not involve with the manuscript preparation and its findings. The authors declare that they have no conflicts of interest.
