# Supplementary material for: Reliability and validity of time-use surveys in assessing 24-hour movement behaviors in adults
Source: J Exerc Sci Fit. 2025 Mar 22;23(2):133–40. doi: 10.1016/j.jesf.2025.03.003 (PMC11986218; doi:10.1016/j.jesf.2025.03.003)
Supplement: Multimedia component 1 [file mmc1.docx]

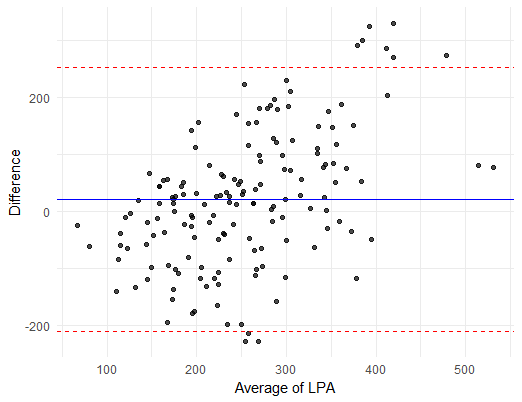

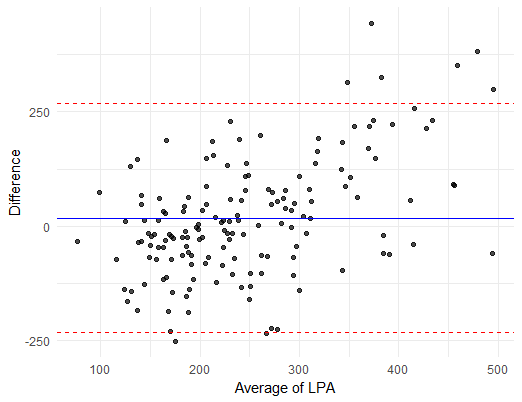

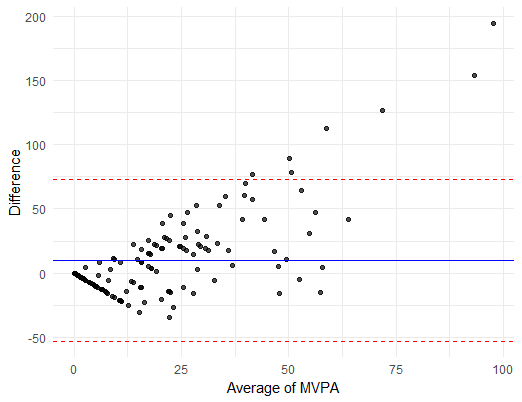

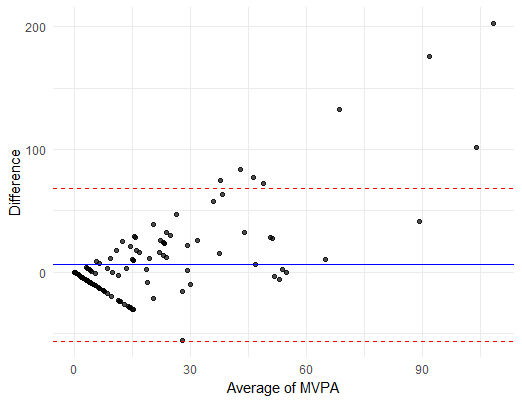

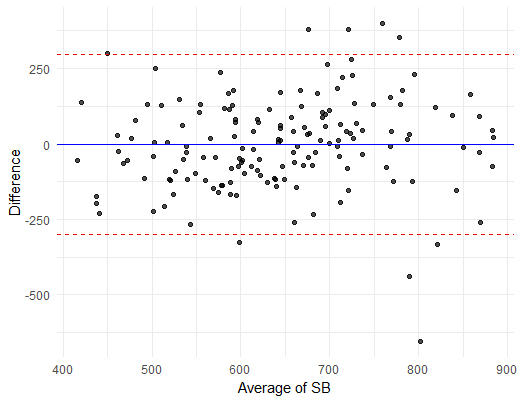

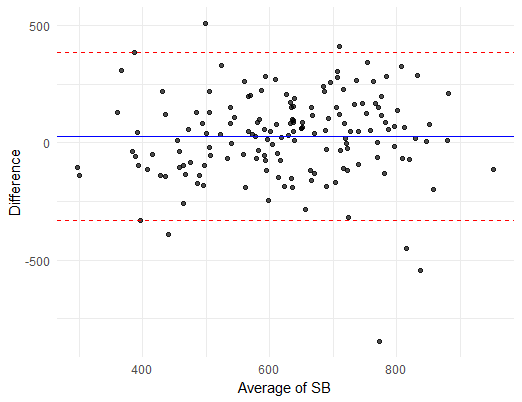


**Figure 1a**

**Figure 1b**

**Figure 1c**

**Figure 1d**

**Figure 1e**

**Figure 1f**

**Figure 1.** Bland-Altman Plots for Agreement Between ICATUS-Based Time-Use Estimates and Accelerometer-Derived Measures Across Two Measurement Periods

**Notes**: Bland-Altman plots illustrate the agreement between ICATUS-based time-use estimates and accelerometer-derived measures for **sedentary behavior (SB), light physical activity (LPA), and moderate-to-vigorous physical activity (MVPA)** across two measurement periods: **Days 2 vs. 9 (Panels a–c) and Days 3 vs. 10 (Panels d–f).** The solid blue line represents the mean difference (bias), and the dashed red lines indicate the **95% limits of agreement (LOA).**
